# WHY DIDN’T YOU DO THAT TODAY?: EXPLANATIONS FOR INCOMPLETE INTENDED ACTIVITIES RARELY INCLUDE COGNITION

**DOI:** 10.1093/geroni/igae098.1978

**Published:** 2024-12-31

**Authors:** Jacqueline Mogle, Nikki Hill, Erin Harrington, Jennifer Turner, Jody Nicholson, Robert Stawski

**Affiliations:** Clemson University, Clemson, South Carolina, United States; The Pennsylvania State University, University Park, Pennsylvania, United States; University of Wyoming, Laramie, Wyoming, United States; University of Hawaii Hilo, Hilo, Hawaii, United States; Clemson University, Clemson, South Carolina, United States; University of Essex, Colchester, England, United Kingdom

## Abstract

Completing intended activities, such as household chores, relies on a variety of cognitive processes to plan and execute within our typical daily routine. However, when we fail to complete an intended activity, explanations for the lack of completion can span reasons that are explicitly (e.g., couldn’t concentrate) to implicitly (e.g., not feeling well) related to cognitive functioning. The current study used daily diaries to examine individuals’ explanations for not completing intended activities. Two hundred forty-six community-dwelling adults (Mage=49.1, range 20-79 years) completed evening reports in a 7-day diary study (Mdiaries=6.2). We examined whether likelihood of not completing any of eight intended tasks (e.g., physical activity, household chores, work-related tasks) and participant’s explanations for the lack of completion (e.g., couldn’t concentrate, forgot, not enough time) depended on age. Participants reported an incomplete intended activity on 49% of days (n=535) and the most common reason was not enough time (27%). Multilevel logistic regression revealed older adults were more likely to report incomplete intended activities compared to younger (OR=1.72, CI: 1.25-2.37) and to indicate lack of time (OR=1.57, CI: 1.14-2.18) or needing to complete something more important (OR=1.67, CI: 1.18-2.37). Reasons explicitly involving cognitive processes were reported on 9% of incomplete activities and did not differ across age (p=.99). These results provide insight into ways older adults may explain not completing activities without implicating difficulties in their cognitive functioning. We will discuss the explicit role of cognition in explanations individuals use that implicitly implicate cognitive difficulties to improve methods for assessing cognitive functioning.

